# Schiff-Base Crosslinked Stretchable Multifunctional Hydrogel Enables Combined Therapy in Diabetic Wound Healing

**DOI:** 10.3390/jfb17100493

**Published:** 2026-09-30

**Authors:** Ze-Dong Cai, Yao-Wen Zhang, Xin-Ru Zou, Jia Ju, Chao Fang, Ming-Kai Li, Bang-Le Zhang

**Affiliations:** 1 Department of Pharmaceutics, School of Pharmacy, and Key Laboratory of Pharmacology of the State Administration of Traditional Chinese Medicine, Fourth Military Medical University, Xi’an 710032, China; pharmczd@163.com (Z.-D.C.); z17860554048@163.com (Y.-W.Z.); zxr01092024@163.com (X.-R.Z.); 2 Department of Pharmacy, School of Stomatology, Fourth Military Medical University, Xi’an 710032, China; 3 Department of Pharmacology, School of Pharmacy, Fourth Military Medical University, Xi’an 710032, China; fang1989@fmmu.edu.cn (C.F.); mingkai@fmmu.edu.cn (M.-K.L.)

**Keywords:** diabetic wound healing, Schiff-base crosslinked hydrogel, combined therapy, antibacterial activity, tissue regeneration

## Abstract

Diabetic wound healing remains a challenge due to persistent oxidative stress, defective angiogenesis and bacterial infection. Herein, a multifunctional hydrogel Y/PDA@HAPLL was engineered via Schiff-base crosslinking between oxidized hyaluronic acid (OHA) and ε-poly-L-lysine (ε-PLL) and incorporating Y-27632 (Y, *R*-trans-4-(1-aminoethyl)-*N*-(4-pyridyl) cyclohexanecarboxamide dihydrochloride) and polydopamine (PDA). The formulation was optimized using response surface methodology, thus achieving rapid gelation (3.8 s), strong tissue adhesion (10 kPa) and excellent stretchability. Under 808 nm laser irradiation, Y/PDA@HAPLL exhibited stable photothermal conversion and good antibacterial activity to effectively eradicate the bacterial biofilms and achieve >99% killing of *S. aureus* and *E. coli*. In vitro, skin re-epithelialization (keratinocyte proliferation and migration) and endothelial repair (tube formation) under high glucose were promoted by Y/PDA@HAPLL. In the diabetic mice with infected wounds, Y/PDA@HAPLL significantly accelerated wound closure and tissue regeneration by combining PTT antibacterial activity, ROS scavenging, endothelial repair, angiogenesis, and re-epithelialization, while concurrently downregulating ROCK1 and p-DRP1, upregulating p-eNOS and CD31, reducing mtROS, and promoting collagen deposition and remodeling. In addition, no organ damage and hepatorenal toxicity were observed during treatment period. Taken together, Y/PDA@HAPLL provided a multifunctional paradigm for the combined therapy of infected diabetic wounds by integrating rapid gelation, PTT antibacterial activity, and regeneration promotion.

## 1. Introduction

Currently, diabetes mellitus is one of the most common chronic metabolic conditions seen worldwide. Globally, the International Diabetes Federation states that the diabetic population has crossed 500 million and keeps climbing annually [1,2,3]. Among the various complications, diabetic wounds—particularly diabetic foot ulcers—represent one of the most common and severe consequences, affecting approximately 15–25% of diabetic patients during their lifetimes, with up to 85% of these individuals facing a high risk of limb amputation [4,5,6]. The resulting physical disability, psychological distress, and socioeconomic burden underscore an urgent need for effective therapeutic strategies [7,8,9].

The refractory nature of diabetic wounds arises from a complex interplay of pathological factors, among which bacterial infection is a primary driver of healing impairment [10,11]. Pathogenic bacteria colonize wound surfaces to form biofilms that confer remarkable resistance to conventional antibiotics and host immune clearance [12,13]. The growing prevalence of multidrug-resistant bacteria further compounds this therapeutic challenge, underscoring an urgent need for non-antibiotic antimicrobial strategies [14,15]. Photothermal therapy (PTT) employs photothermal agents to generate localized hyperthermia upon near-infrared (NIR) irradiation and has emerged as a promising alternative antimicrobial strategy [16,17,18]. Polydopamine (PDA), synthesized through the self-polymerization of dopamine, has emerged as a versatile functional material that combines outstanding photothermal antibacterial activity, strong tissue adhesion, and free radical scavenging activity for infected wounds [17,19]. Unlike traditional antibiotics, PTT is able to overcome bacterial resistance and exhibits broad-spectrum antibacterial effects on both Gram-positive and Gram-negative pathogens [19,20,21].

Beyond infection control, successful wound repair requires the orchestration of hemostasis, inflammation, proliferation, and tissue remodeling, where angiogenesis is a critical rate-limiting step for granulation tissue formation and re-epithelialization [22,23]. However, in the diabetic microenvironment, persistent hyperglycemia-driven oxidative stress and inflammation severely compromise this angiogenic cascade [24,25]. Accumulating evidence has identified the aberrant activation of Rho-associated coiled-coil containing protein kinase 1 (ROCK1) as a central molecular hub in diabetes-impaired angiogenesis [26,27]. Hyperglycemia markedly upregulates ROCK1 expression in wound granulation tissues [28]. Activated ROCK1 not only directly inhibits AMPKα phosphorylation at Thr172 to block the AMPK/eNOS/NO signaling axis, leading to reduced nitric oxide production and endothelial dysfunction, but also forms a protein complex with RIPK4 to relieve the inhibition of DRP1 phosphorylation at Ser616 [29]. This triggers excessive mitochondrial fission and a burst of mitochondrial reactive oxygen species (mtROS), culminating in severe oxidative stress and endothelial apoptosis [30]. This molecular cascade ultimately results in microcirculatory impairment and failure of vascular network reconstruction [31]. Therefore, inhibition of ROCK1 has emerged as a promising strategy to restore endothelial proliferation, migration, and tube formation. Y-27632, as a highly selective ROCK1 inhibitor, has shown potential in promoting wound healing and angiogenesis [32,33].

In recent years, hydrogel-based wound dressings have garnered considerable attention as effective therapeutic platforms [34,35,36]. Hydrogels possess the unique combination of properties, including high water content, tissue-like softness, excellent biocompatibility, tunable mechanical properties, and the ability to deliver bioactive agents in a controlled manner, that make them exceptionally suited for wound healing applications [37,38]. As a naturally occurring glycosaminoglycan and a major component of the extracellular matrix, hyaluronic acid (HA) stands out as an ideal hydrogel building material, which exhibits exceptional biocompatibility, biodegradability, and abundant chemically modifiable functional groups [39,40,41]. Oxidized hyaluronic acid (OHA), bearing aldehyde groups, can form dynamic Schiff-base linkages with amino-containing materials, enabling rapid in situ gelation and strong tissue adhesion [42,43].

**Scheme 1:**
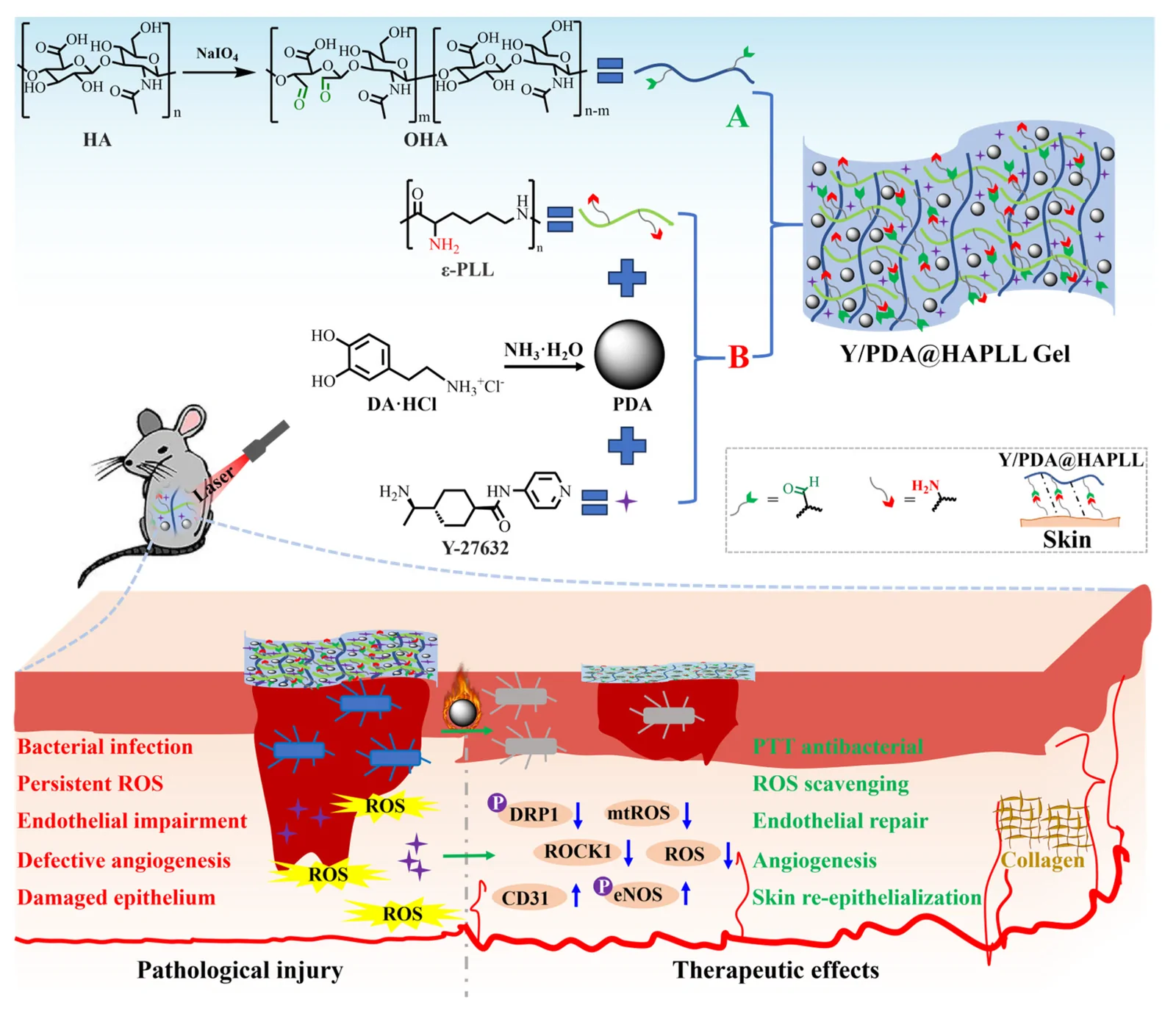
Illustration of the design of multifunctional hydrogel system Y/PDA@HAPLL for the combined treatment of infected diabetic wounds.

Herein, a multifunctional hydrogel system Y/PDA@HAPLL was designated via Schiff-base crosslinking between OHA and ε-polylysine (ε-PLL) with rich amino groups [44,45], and incorporating the ROCK1 inhibitor Y-27632 (Y) and polydopamine (PDA) for the combined treatment of infected diabetic wounds (Scheme 1). The design was guided by several key considerations: (i) the OHA/ε-PLL Schiff-base network enables rapid in situ gelation and strong tissue adhesion; (ii) the incorporation of PDA confers photothermal antibacterial activity upon NIR laser irradiation; (iii) the sustained release of Y-27632 from the Schiff-base network hydrogel matrix can downregulate mtROS levels, suppress endothelial apoptosis, promote cell proliferation and migration, and upregulate p-eNOS expression, thereby restoring endothelial function and inducing neovascularization. The formulation of Y/PDA@HAPLL was optimized using response surface methodology, and the physicochemical properties of the Y/PDA@HAPLL hydrogel, including gelation kinetics, rheological behavior, mechanical properties, tissue adhesion, self-healing capability, and photothermal conversion performance were systematically characterized. A series of in vitro and in vivo assessments were performed to further evaluate the biocompatibility, antibacterial activity, and combined wound healing effects on infected skin wounds in the diabetic mice. Through the multifunctional paradigm of photothermal sterilization and pro-angiogenic activity, Y/PDA@HAPLL provided a promising strategy to address the key pathological barriers that impede diabetic wound healing.

## 2. Materials and Methods

### 2.1. Materials

Sodium hyaluronate (HA, Mw 35 kDa) was obtained from Shanghai Yuanye Biotechnology Co., Ltd. (Shanghai, China). Sodium periodate and ethylene glycol were obtained from Shanghai Zhanyun Chemical Co., Ltd. (Shanghai, China) and Shanghai Aladdin Biochemical Technology Co., Ltd. (Shanghai, China), respectively. Dopamine hydrochloride was procured from Sain Chemical Technology Co., Ltd. (Shanghai, China), and ε-polylysine (ε-PLL, Mw < 5000 Da) hydrochloride was acquired from Macklin Chemical Co., Ltd. (Shanghai, China). Y-27632 was purchased from Beyotime Biotechnology Co., Ltd. (Shanghai, China). Streptozotocin was purchased from Macklin Biochemical Co., Ltd. (Shanghai, China). Dulbecco’s modified Eagle’s medium (DMEM) was purchased from HyClone (Logan, UT, USA), and fetal bovine serum (FBS) was supplied by ExCell Bio Co., Ltd. (Shanghai, China). LB agar plates were obtained from Biosharp (Hefei, China). The live/dead cell staining kit was purchased from APExBIO Technology LLC (Shanghai, China). All experimental animals were obtained from the Fourth Military Medical University (Xi’an, China), and all procedures were performed in accordance with the guidelines of the Animal Ethics Committee of the Fourth Military Medical University (approval No. IACUC-20250099).

### 2.2. Preparation and Characterization of Materials Used in Hydrogels

#### 2.2.1. Preparation and Characterization of Oxidized Hyaluronic Acid (OHA)

Oxidized hyaluronic acid (OHA) was synthesized based on a reported protocol with minor adjustments [42,46]. Sodium hyaluronate (2 g, HA) was dissolved in 100 mL of deionized water to obtain a 2% (*w*/*v*) aqueous solution. Sodium periodate (2.3 g) was dissolved in 10 mL of deionized water and then slowly added to the HA aqueous solution under dark conditions. The resulting mixture was stirred at room temperature (RT) for 4 h. After the addition of ethylene glycol (5 mL), the solution was dialyzed against deionized water for 48 h with the water being refreshed every 8 h and then frozen at −80 °C and lyophilized to constant weight, yielding the aldehyde-modified OHA. The structure and oxidation degree of OHA was characterized by ^1^H NMR spectroscopy (Bruker Biospin, Fällanden, Switzerland).

#### 2.2.2. Preparation and Characterization of Polydopamine (PDA)

Ammonia aqueous solution (1.8 mL), ethanol (24 mL), and deionized water (54 mL) were mixed and stirred for 10 min. A solution of dopamine hydrochloride (300 mg) in deionized water (6 mL) was then slowly added, and the mixture was stirred at RT for 24 h and subsequently centrifuged at 15,000× *g* for 10 min at 4 °C. The supernatant was discarded, and the obtained pellet was washed three times with deionized water. The resulting PDA were characterized by dynamic light scattering (DLS, Malvern Panalytical Ltd., Malvern, UK), transmission electron microscopy (TEM, Thermo Fisher Scientific, Hillsboro, OR, USA), and scanning electron microscopy (SEM, Thermo Fisher Scientific, Hillsboro, OR, USA).

### 2.3. Preparation and Characterization of Hydrogels

#### 2.3.1. Preparation and Gelation Time Measurement

The hydrogel was prepared via an aldehyde–amine Schiff-base crosslinking reaction. OHA powders were dissolved in 0.3 mol/L disodium hydrogen phosphate (DSP) at concentrations of 10%, 15% and 20% (*w*/*v*) and stored at 4 °C in the dark. ε-PLL solutions with corresponding concentrations were prepared separately. Equal volumes of OHA and ε-PLL solutions were mixed, vortexed and incubated at 37 °C. The fluidity of the mixture was monitored by repeated inversion, and the time required to form a stable gel that did not flow upon inversion was recorded as the gelation time.

#### 2.3.2. Adhesion Strength Test

The adhesion performance of the Y/PDA@HAPLL hydrogel was evaluated using a lap shear test on porcine skin. Rectangular skin specimens were stored in saline at 4 °C and preheated at 37 °C for 30 min prior to testing. A 100 µL aliquot of the hydrogel precursor was coated onto one specimen, which was then overlapped with another specimen to create a bonding area of 10 × 10 mm and pressed for 30 s. The bonded samples were pulled at a crosshead speed of 5 mm/min using a universal testing machine equipped with a 50 N load cell until complete separation (Model 68TM-5, Instron Corporation, Norwood, MA, USA). The maximum force was recorded, and the adhesion strength was calculated as the force divided by the bonding area.

#### 2.3.3. Formulation Optimization of Hydrogel

To optimize the hydrogel formulation for the application to encapsulate PDA and Y-27632, gelation time and adhesion strength were selected as key response variables using response surface methodology (RSM). A two-factor, three-level central composite design (CCD) was employed, with OHA (10%, 15% and 20%) and ε-PLL (10%, 25% and 40%) as independent variables. Nonlinear regression models were constructed using Design-Expert software (version 12.0, Stat-Ease, Inc., Minneapolis, MN, USA), and the significance of the models was verified by analysis of variance (ANOVA). The optimal formulation was determined based on the criteria of rapid gelation and stable adhesion strength.

#### 2.3.4. Preparation of the Optimal Y/PDA@HAPLL Hydrogel

Solution A was prepared by dissolving OHA (19.35%, *w*/*v*) in 0.3 mol/L DSP. Solution B consisted of ε-PLL (22.29%, *w*/*v*) with Y-27632 (0.16 mg/mL) and PDA (0.5 mg/mL). Equal volumes of solutions A and B were blended and vortexed to form the hydrogel network that encapsulated both PDA and Y-27632.

#### 2.3.5. Morphological Characterization

The morphology of Y/PDA@HAPLL and HAPLL hydrogels was examined using field emission scanning electron microscopy (FESEM). Briefly, lyophilized samples were prepared by freezing at −50 °C for 24 h under a pressure of <10 Pa to remove moisture. The dried samples were then fractured in liquid nitrogen for 5 min, sputter-coated with gold, and imaged to observe pore structure, pore size distribution, and surface compactness.

#### 2.3.6. Rheological Measurement

A rotational rheometer (20 mm parallel plate, 0.5 mm gap, Malvern Panalytical Ltd., Malvern, UK) was used to determine rheological properties at 37 °C, following standard procedures. After mixing, the precursor solutions were immediately loaded onto the plate, excess sample was trimmed, and a silicone oil seal was applied to prevent water evaporation. Time-sweep tests were conducted to monitor the storage modulus (G′) and loss modulus (G″) during the gelation process. Additionally, strain sweep tests and alternating step-strain tests were performed to evaluate the viscoelastic stability and mechanical performance of the hydrogels.

#### 2.3.7. Adhesion, Self-Healing Ability and Stretchability Evaluation

The adhesion and mechanical flexibility of Y/PDA@HAPLL on porcine skin were further assessed through adhesion, bending, twisting and squeezing tests. Self-healing ability was evaluated by cutting cylindrical hydrogel samples into two pieces, aligning the cut surfaces, allowing them to rest for 5 min, and verifying the recovery via laser observation and skin adhesion tests. Stretchability was examined by uniformly stretching the hydrogels to record deformation and elongation at break, thereby assessing their clinical mechanical adaptability.

#### 2.3.8. Drug Release Assay

The Y/PDA@HAPLL hydrogel samples (10 mg) were immersed in 1 mL of PBS (pH 7.4 and pH 4.5, respectively) and incubated at 37 °C with shaking at 100 rpm. At scheduled time points, the supernatant (100 μL) was sampled and centrifuged for high performance liquid chromatography (HPLC) detection. Meanwhile, 100 μL of fresh PBS was replenished to maintain a constant volume. The released Y (rn) was quantified using an Agilent 1260 Infinity HPLC system (Agilent Technologies, Santa Clara, CA, USA) equipped with a Thermo C18 column (Thermo Fisher Scientific, Waltham, MA, USA). The mobile phase was methanol/0.1% trifluoroacetic acid (TFA) aqueous solution (25:75, *v*/*v*) at 1.0 mL/min, with a column temperature of 25 °C, detection wavelength of 244 nm, and injection volume of 10 μL. The cumulative release rate of Y (r_n_) was calculated using the following formula:

$$r_{n}=\frac{1c_{n}+0.1\times \sum_{i=1}^{n-1}c_{i}}{m_{\mathrm{initial}}}\times 100\%$$

In this equation, *m_initial_* is the amount of initially added Y. *c_n_* and *c_i_* are the concentration of Y in the *n*th and *i*th collection times.

#### 2.3.9. Photothermal Conversion Performance Evaluation

The photothermal conversion performance of HAPLL, Y@HAPLL, PDA@HAPLL, Y/PDA@HAPLL, and a phosphate-buffered saline (PBS) control was investigated. A laser probe (Model 808, Changchun New Industries Optoelectronics Tech. Co., Ltd., Changchun, China) and a thermal imager (FLIR E8, FLIR Systems, Inc., Wilsonville, OR, USA) were fixed at distances of 10 cm and 30 cm, respectively. For power-dependence analysis, Y/PDA@HAPLL was irradiated at gradient power densities, and thermal images along with temperature–time curves were recorded every 30 s over a 5 min period. For formulation comparison, PBS, HAPLL, Y@HAPLL, PDA@HAPLL, and Y/PDA@HAPLL were irradiated at 1.0 W/cm^2^, with temperature changes recorded at 30 s intervals for 5 min, generating dynamic heating curves and corresponding thermal images for each group.

### 2.4. In Vitro Cytocompatibility Evaluation

#### 2.4.1. MTT Assay

The cytocompatibility of the Y/PDA@HAPLL hydrogel was evaluated using an MTT assay. Hydrogel samples were sterilized by UV irradiation for 30 min. They were then immersed in complete DMEM (400 mg/mL) supplemented with 10% FBS and 1% P/S. Incubation followed at 37 °C under 5% CO_2_ for 24 h. The resulting extract was then filtered through a 0.22 µm pore-size membrane and stored at 4 °C in the dark. NIH3T3 cells were placed in 96-well plates at a seeding density of 5 × 10^3^/well and grown for 24 h. The cells were subsequently treated with 100 µL of various dilutions of the hydrogel extract for 24 h, with blank control cells receiving only fresh medium. After removal of the extract, 20 μL of MTT solution (5 mg/mL) was added to each well, followed by an additional 4 h of incubation. The medium was carefully aspirated, and 150 μL of DMSO was added to each well, and the plates were shaken for 10 min. The optical density was measured at 450 nm (OD_450_), and cell viability was calculated relative to the blank control group.

#### 2.4.2. Live/Dead Cell Staining Assay

Cytocompatibility was further assessed using a live/dead cell staining kit. NIH3T3 cells were seeded into 24-well plates at a density of 1 × 10^4^ cells per well in 500 µL of complete DMEM (10% FBS, 1% P/S) and cultured for 24 h. The culture medium was then replaced with 500 µL of hydrogel extracts at various concentrations, while blank control wells received fresh medium only. Following incubation for 24 or 48 h, the supernatants were discarded, and the cells were washed with PBS. The staining working solution was first prepared as per the manufacturer’s manual. It was then added to each well and incubated for 15–20 min in the absence of light. Fluorescence images were subsequently captured with a fluorescent microscope.

#### 2.4.3. In Vitro Hemolysis Test

The blood compatibility of HAPLL, Y@HAPLL, PDA@HAPLL, and Y/PDA@HAPLL was evaluated by a hemolysis assay. Hydrogel extracts were prepared by immersing the samples (200 mg/mL) in saline at 37 °C for 24 h. After filtration, the extracts were stored at 4 °C. Rat red blood cells (RBCs) were collected by centrifugation, washed, and diluted to 2% (*v*/*v*) in saline. The test groups, including HAPLL, Y@HAPLL, PDA@HAPLL, and Y/PDA@HAPLL, were mixed with the RBC suspension at a ratio of 1:9 (*v*/*v*). Saline and deionized water were used as negative and positive controls, respectively. All tubes were incubated at 37 °C for 3 h, after which photographs were taken, and the tubes were centrifuged. The absorbance of the supernatant was measured at 540 nm. The hemolysis rate was calculated based on the absorbance values relative to the control.

$$\mathrm{Hemolysis rate}\left(\%\right)=\frac{\left({\mathrm{OD}}_{\mathrm{sample}}-{\mathrm{OD}}_{\mathrm{negative}}\right)}{\left({\mathrm{OD}}_{\mathrm{positive}}-{\mathrm{OD}}_{\mathrm{negative}}\right)}\times 100\%$$

### 2.5. In Vitro Antibacterial Activity Evaluation

#### 2.5.1. Effect on Bacterial Colony Formation

The antibacterial performance of the hydrogels against *Staphylococcus aureus* (*S. aureus*, CGMCC, Beijing, China) and *Escherichia coli* (*E. coli*, CGMCC, Beijing, China) was assessed using a colony formation assay. For groups containing PDA nanoparticles, an 808 nm laser (1 W/cm^2^, 5 min) was applied to achieve photothermal effects. Hydrogels (10 mm in diameter) were placed in 48-well plates, and 100 µL of bacterial suspension (1 × 10^6^ CFU/mL) along with 100 µL of Luria–Bertani (LB) medium were added to each well. The plates were pre-incubated at 37 °C for 15 min, during which the laser groups were irradiated. Subsequently, 800 µL of LB medium was added, and the plates were incubated at 37 °C for 18 h. The resulting bacterial suspensions were diluted 1000-fold, plated onto LB agar, and incubated for an additional 18 h. The number of colonies was counted, and the antibacterial efficiency was calculated.

#### 2.5.2. The Zone of Inhibition Assay

Antibacterial activity against *S. aureus* and *E. coli* was further evaluated using the agar well diffusion method, with photothermal effect applied to PDA-containing groups under 808 nm laser irradiation (1 W/cm^2^, 5 min). Bacterial lawns were prepared on LB agar plates, and hydrogel disks (6 mm diameter, 2 mm height) were placed onto the agar surface. PBS-soaked filter paper disks were used as controls. The PDA groups were subjected to laser irradiation, while non-irradiated counterparts served as additional controls. After 18 h of incubation at 37 °C, the inhibition zone diameters on all plates were quantified to evaluate the antibacterial effect.

#### 2.5.3. Effect on Mature Bacterial Biofilm

The biofilm eradication performance of the hydrogels against *S. aureus* and *E. coli* was evaluated via a crystal violet staining assay. For groups containing PDA nanoparticles, an 808 nm laser (1 W/cm^2^, 5 min) was used for irradiation to achieve photothermal antibiofilm effects. Mature bacterial biofilms were pre-formed by incubating bacterial suspension (1 × 10^6^ CFU/mL) in 48-well plates at 37 °C for 24 h. The hydrogel samples (10 mm in diameter) were placed into each well, and the laser irradiation was performed for photothermal treatment groups. After co-incubation at 37 °C for 18 h, the non-adherent planktonic bacteria were removed by gentle PBS washing. The residual biofilms were fixed with methanol and stained with crystal violet solution, and the plate was photographed before the crystal violet was fully dissolved with 33% glacial acetic acid. The absorbance values were measured using a microplate reader, and the biofilm eradication efficiency was calculated accordingly.

#### 2.5.4. Effect on Bacterial Morphology

Morphological changes in bacteria after treatment were observed using FESEM to elucidate the antibacterial mechanisms. The PDA groups were irradiated under 808 nm laser (1 W/cm^2^, 5 min), with non-irradiated and PBS groups serving as controls. Bacteria were co-cultured with PBS, HAPLL, Y@HAPLL, PDA@HAPLL, and Y/PDA@HAPLL at 37 °C for 18 h, after which the bacterial cells were collected by centrifugation, washed, fixed, dehydrated, dried, and sputter-coated with gold. The specimens were then imaged by FESEM to assess structural damage to the bacterial cells.

### 2.6. In Vitro Wound Healing Properties

#### 2.6.1. Cell Proliferation Experiment

The wound healing performance of the hydrogels was evaluated using the MTT assay on HaCaT cells. Hydrogel extracts (50 mg/mL) from the HAPLL, Y@HAPLL, PDA@HAPLL, and Y/PDA@HAPLL groups were added to adherent HaCaT cells seeded in 96-well plates and incubated for 48 h. Following incubation, the cells were treated with the MTT solution for 4 h in the dark, and the resulting formazan crystals were dissolved in DMSO. The optical density was measured at 450 nm (OD_450_), and cell proliferation viability was calculated relative to the control group.

#### 2.6.2. Cell Migration Experiment

The cell migration-promoting effect was evaluated using a wound healing scratch assay. HaCaT cells were cultured to confluence in 6-well plates, and a linear scratch was created across the monolayer using a pipette tip. The wells were rinsed with PBS and then treated with serum-free medium containing 50 mg/mL of the respective hydrogel extracts (HAPLL, Y@HAPLL, PDA@HAPLL, and Y/PDA@HAPLL). Following 24 h and 48 h incubations, the cells were labeled with a live-cell dye, and the scratch areas were observed and quantified using fluorescence microscopy (Model BX53, Olympus Corporation, Tokyo, Japan) to assess the wound healing capacity.

#### 2.6.3. In Vitro Pro-Angiogenesis Experiment

The pro-angiogenic performance under hyperglycemic conditions was assessed using a tube formation assay with human umbilical vein endothelial cells (HUVECs). Matrigel was solidified in 24-well plates. The experimental groups included normal medium, high-glucose medium (25 mmol/L), and high-glucose medium supplemented with 50 mg/mL extracts of HAPLL, Y@HAPLL, PDA@HAPLL, or Y/PDA@HAPLL. HUVECs were seeded onto the Matrigel and incubated for 12 h. The cells were then stained with a live-cell dye and imaged by fluorescence microscopy to observe the tubular structures.

### 2.7. In Vivo Activity Evaluation

#### 2.7.1. Photothermal Conversion Evaluation

The in vivo photothermal conversion performance of the normal saline, HAPLL, Y@HAPLL, PDA@HAPLL, and Y/PDA@HAPLL was evaluated in six-week-old male C57BL/6J mice. Full-thickness dorsal wounds (6 mm in diameter) were covered with the hydrogels and irradiated with an 808 nm laser (1 W/cm^2^, 5 min). Thermal images were recorded every 30 s (laser probe at 10 cm, thermal imager at 30 cm) to assess the photothermal conversion efficiency.

#### 2.7.2. Therapeutic Evaluation on Infected Diabetic Wound

The in vivo therapeutic efficacy of normal saline, HAPLL, Y@HAPLL, PDA@HAPLL, and Y/PDA@HAPLL against infected diabetic wounds was systematically evaluated in a murine diabetic wound model. Mice were housed in individually ventilated cages under constant-temperature conditions with an 11 h light–dark cycle (lights on 07:00–18:00). Cage positions on the housing rack were re-randomized every three days to minimize positional bias. Environmental enrichment, including bedding material for nesting and gnawing sticks, was provided within each cage. Standard laboratory chow and tap water were provided. Briefly, six-week-old male C57BL/6J mice were fed a high-fat and high-sugar diet (60% kcal from fat, Shuyu Biotechnology (Shanghai) Co., Ltd., Shanghai, China) for six consecutive weeks to induce insulin resistance. After that, streptozotocin (STZ) was administrated by intraperitoneal injection, 40 mg/kg per day, for 5 days in a row. At 7 days after the final STZ dose, fasting blood glucose (FBG) levels were evaluated (Sinocare Inc., Changsha, China), and mice exhibiting stable FBG levels ≥ 11.1 mmol/L (200 mg/dL) were considered successfully established as diabetic mice. The diabetic mice were anesthetized via isoflurane inhalation, and the dorsal hair was thoroughly removed. A standardized full-thickness circular skin wound (6 mm in diameter) was created on the dorsal skin using a sterile biopsy punch. To establish a clinically relevant infected diabetic wound model, 100 µL of a *S. aureus* suspension (1 × 10^6^ CFU/mL) was uniformly applied to each wound surface. The wounds were then covered with sterile plastic film for 24 h to promote bacterial biofilm formation.

The aforementioned mice were allocated randomly to five groups (six per group): normal saline, HAPLL, Y@HAPLL, PDA@HAPLL, and Y/PDA@HAPLL. During the 14-day treatment period, body weight and wound photographs were recorded on day 1, 4, 7, 10, and 13, and the corresponding hydrogel dressings were replaced. The wound healing rate for each group was quantitatively determined based on the dynamic changes in wound area. On day 14 post-treatment, orbital venous blood was collected for serum biochemical analysis to evaluate hepatic and renal function. All wound skin tissues were excised for bacterial colony counting and histopathological staining analyses. The staining panel included hematoxylin-eosin (H&E) staining, Masson staining, and immunofluorescence staining for ROCK1, p-DRP1, p-eNOS, mtROS, and CD31. In addition, major organs (heart, liver, spleen, lung, and kidney) were harvested for histological examination to assess the potential organ toxicity.

### 2.8. Statistical Analysis

All data are expressed as mean ± SD. Normality and homogeneity of variances were assessed using the Shapiro–Wilk test and Levene’s test, respectively. The sample size (n = 6 per group) was determined via power analysis (α = 0.05, power = 0.8), based on published effect sizes from diabetic wound healing studies (expected 30% difference, SD = 15%). One additional mouse was reserved per group to account for potential surgical attrition in accordance with the 3R principle. For longitudinal repeated wound area measurements, a two-way repeated-measures ANOVA (group × time) was performed, incorporating the Greenhouse–Geisser correction for sphericity, followed by Bonferroni post hoc tests. One-way ANOVA or an unpaired *t*-test was adopted for comparisons at single time points. All statistical analyses were conducted in GraphPad Prism 9.0 (GraphPad Software, Inc., San Diego, CA, USA), and figures were plotted using Origin 2024 (OriginLab Corporation, Northampton, MA, USA). Statistical significance was defined as * *p* < 0.05, while ** *p* < 0.01 was considered highly significant.

## 3. Results and Discussion

### 3.1. Preparation and Characterization of Hydrogel

#### 3.1.1. Preparation and Characterization of Hydrogel Materials

OHA was obtained as a white solid with 95% yield via periodate oxidation. Compared with HA, three new chemical shifts at 4.9–5.1 ppm appeared in the ^1^H NMR spectrum of OHA, which were assigned to methylene protons derived from oxidized aldehyde groups. The two adjacent aldehyde groups readily formed acetal with abundant hydroxyl groups on HA chains, which accounted for the observed signals at 4.9–5.1 ppm (Figure S1). The oxidation degree of OHA was calculated to be 19% by ^1^H NMR spectroscopy [42,46]. PDA nanoparticles were prepared through alkali-induced oxidative self-polymerization of dopamine (Figure S2). Dynamic light scattering (DLS) revealed a narrow size distribution with a mean hydrodynamic diameter of approximately 160 nm (Figure S3), and TEM as well as SEM images confirmed a regular spherical morphology (Figure S4). Such uniform size and well-defined morphology are favorable for homogeneous incorporation into the hydrogel matrix and for ensuring consistent photothermal conversion performance, as particle size directly influences near-infrared absorption efficiency.

#### 3.1.2. Optimization of Hydrogel by Response Surface Methodology

Response surface methodology (RSM) was employed to optimize the formulation of the OHA/ε-PLL hydrogel. A two-factor, three-level central composite design was applied, with OHA concentrations (10%, 15% and 20%) and ε-PLL concentrations (10%, 25% and 40%) as independent variables, and gelation time and adhesion strength as response variables (Table S1 and Table S2). Nonlinear regression models were constructed using Design-Expert software (version 12.0), and the significance of the models were validated by regression analysis and analysis of variance (ANOVA) (Tables S3 and S4). According to ANOVA, both models exhibited high significance (** *p* < 0.0001), while the lack-of-fit was non-significant (*p* > 0.05), confirming the excellent reliability and fit of the models. Based on the F-values, the OHA concentration exerted a more prominent effect on both gelation time and adhesion strength than the ε-PLL concentration. The coefficient of determination (*R*^2^) for the gelation time model was 0.9911, while that for the adhesion strength model was 0.9863, and the coefficients of variation (C.V.%) were 8.62% and 6.00%, respectively, indicating outstanding repeatability and stability of the established models.

**Figure 1:**
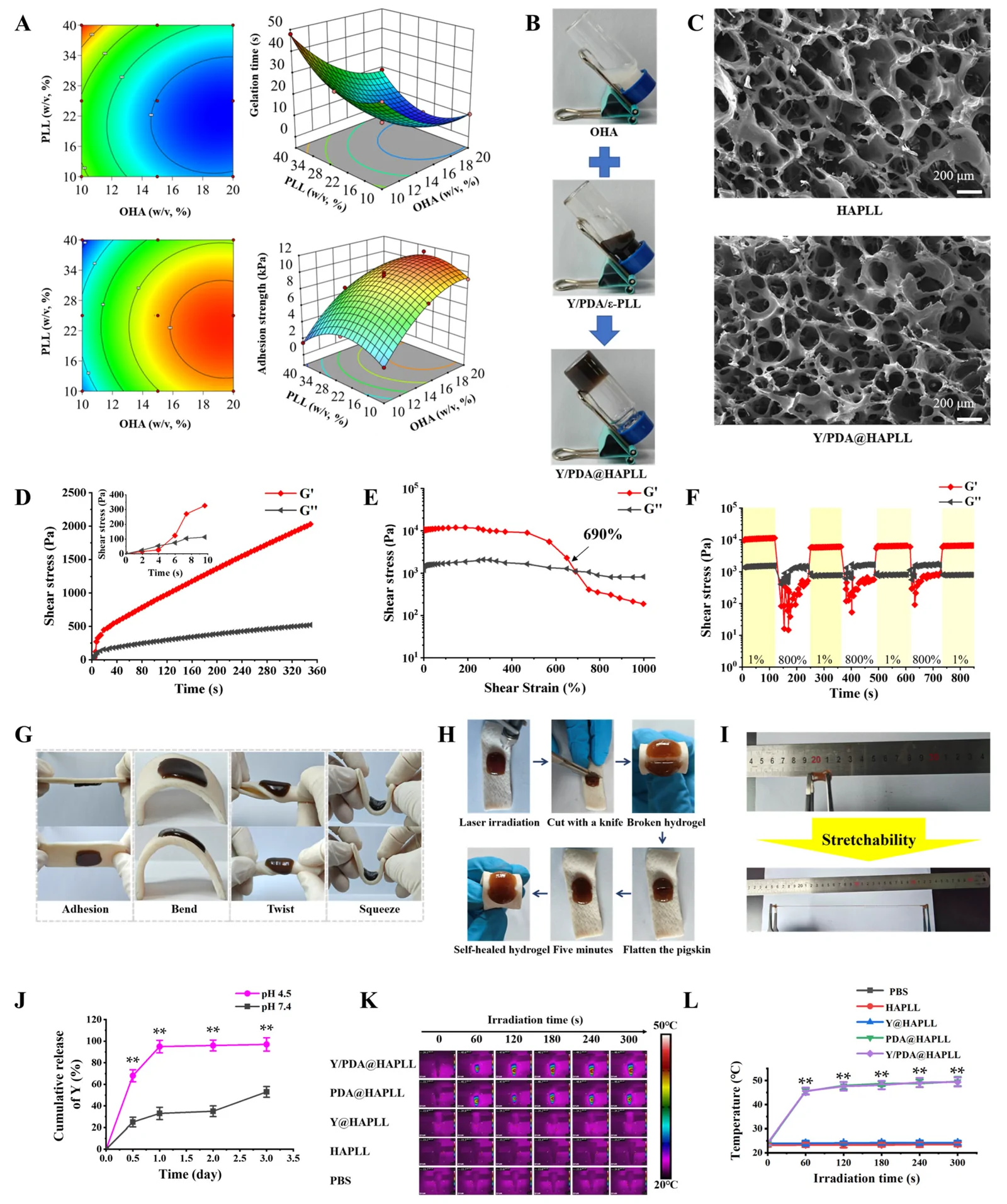
Preparation and characterization of Y/PDA@HAPLL hydrogel. (**A**) Contour (**left**) and response surface (**right**) plots for gelation time (**upper**) and adhesion strength (**lower**). (**B**) Photograph of Y/PDA@HAPLL hydrogel formation. (**C**) SEM images of HAPLL and Y/PDA@HAPLL hydrogel, scale bar = 200 μm. (**D**) Time-sweep test curves recording storage G′ and loss G″ moduli during gelation. (**E**) Strain sweep test revealing the fracture strain of 690%. (**F**) Alternating step-strain test to confirm the rapid self-healing property. (**G**) Digital photos displaying adhesion, bending, twisting and squeezing mechanical performance. (**H**) Macroscopic self-healing demonstration on pigskin tissues after laser cutting. (**I**) Stretchable property of Y/PDA@HAPLL hydrogel. (**J**) Cumulative release of Y from Y/PDA@HAPLL hydrogel (*n* = 3, $\bar{x}$ ± SD). ** *p* < 0.01, compared with the same time group. (**K**) Laser-induced infrared thermal images of different samples. (**L**) Corresponding temperature-change plots over irradiation time (*n* = 3, $\bar{x}$ ± SD). ** *p* < 0.01, compared with the PBS group.

The corresponding three-dimensional response surfaces were presented in Figure 1A. The steep curvature of the surfaces clearly indicated a significant interactive effect between OHA and ε-PLL concentrations, which jointly regulated the hydrogel performance. Within the tested concentration ranges (OHA: 10–20%; ε-PLL: 10–40%), the gelation time sharply decreased with increasing OHA concentration, while it first declined and then increased with rising ε-PLL concentration. In contrast, the adhesion strength continuously increased with OHA concentration but exhibited a unimodal (increase-then-decrease) trend with ε-PLL concentration. Multi-objective optimization using Design-Expert identified the optimal composition as 19.35% OHA and 22.29% ε-PLL, with predicted values of 3.81 s for gelation time and 10.95 kPa for adhesion strength. To verify the predictive accuracy of the model, the optimal formulation was tested in triplicate. The experimentally measured gelation time (3.77 ± 0.47 s) and adhesion strength (10.08 ± 2.02 kPa) were in good agreement with the predicted values (3.81 s and 10.95 kPa, respectively), with no significant difference observed (*p* > 0.05) (Table S5). These results confirmed the reliability and predictive accuracy of the established response surface models, thereby providing a solid foundation for the subsequent construction of the composite hydrogel.

#### 3.1.3. Preparation and Characterization of the Optimized Hydrogel

The optimized Y/PDA@HAPLL hydrogel could undergo rapid in situ gelation as demonstrated in Figure 1B. The gelation occurred within seconds after mixing, forming a cohesive and conformable gel that prevents premature leakage upon injection, which is essential for in situ wound coverage. SEM images (Figure 1C) clearly revealed that the HAPLL hydrogel displayed smooth, irregular pore walls with larger pores, whereas the Y/PDA@HAPLL hydrogel exhibited rougher pore walls but a more uniform and compact network, likely attributable to the incorporation of PDA nanoparticles, which enhanced the crosslinking density and structural homogeneity. The porous structure in the composite is advantageous for cell infiltration and nutrient exchange.

The rheological properties of the hydrogels were systematically evaluated. The time-sweep test (Figure 1D) revealed that the loss modulus (G″) exceeded the storage modulus (G′), indicating a liquid-like state. The crossover point, indicating the sol–gel transition, occurred at 4.5 s, after which G′ remained greater than G″, confirming the formation of a stable three-dimensional network. The strain sweep test (Figure 1E, 0–1000%, 1 Hz) showed that both moduli decreased with increasing strain, with a crossover (G″ > G′) occurring at approximately 690% strain, indicating pronounced shear-thinning behavior. The alternating step-strain test (Figure 1F) demonstrated that upon returning to 1% strain after exposure to high strain, the hydrogel quickly recovered its elastic-dominant state (G″ < G′), and this reversible behavior was reproducible over three cycles. Collectively, these rheological data confirmed that the Y/PDA@HAPLL hydrogel possesses rapid in situ gelation, excellent shear-thinning properties, and a reversible self-healing capability, making it suitable for injection through a syringe, rapid adaptation to irregular wound shapes, and autonomous structural recovery after mechanical disruption—thereby meeting the requirements for in situ wound filling and therapy.

Adhesion tests (Figure 1G) demonstrated that 1 mg of the Y/PDA@HAPLL hydrogel firmly adhered to fresh porcine skin and withstood bending, twisting, and squeezing without detachment or fracture, confirming strong tissue adhesion and excellent mechanical flexibility. Self-healing evaluation (Figure 1H) showed that completely severed hydrogel interfaces fully rejoined after 5 min of contact, and the healed hydrogel endured laser irradiation and skin attachment without re-breaking. Stretchability testing (Figure 1I) revealed remarkable extensibility, with a 2 cm sample elongating beyond 20 cm before rupture. This outstanding deformability, stemming from the dynamic crosslinked network, enabled the hydrogel to adapt to wound tissue movement, preventing cracking or dislodgement, and making it highly suitable for dynamic skin wound repair scenarios. The strong adhesion (10.08 ± 2.02 kPa) is likely facilitated by the abundant aldehyde and catechol groups that form covalent and non-covalent interactions with tissue surfaces, while the self-healing and stretchability arise from the reversible Schiff-base linkages that allow network rearrangement and energy dissipation under stress.

The drug release profiles were evaluated in the acidic diabetic wound microenvironment. As shown in Figure 1J, the Y/PDA@HAPLL hydrogel, with acid-labile Schiff-base linkages, showed pH-dependent release. At pH 4.5, nearly 100% of drug released within 1 day, while at pH 7.4, only ~54% at day 3. The results confirmed rapid drug release in acidic wounds and suppressed leakage under physiological conditions.

The photothermal conversion performance of the Y/PDA@HAPLL hydrogel was evaluated under different laser power densities (0.5, 1.0, 1.2, 1.5 and 2.0 W/cm^2^) over different irradiation duration (Figure S5). The temperature increased with both irradiation time and power density, demonstrating time- and power-dependent photothermal conversion properties. Under 808 nm laser irradiation at 1.0 W/cm^2^, infrared thermal imaging (Figure 1K) and temperature–time curves (Figure 1L) recorded at 30 s intervals revealed that the HAPLL and Y@HAPLL groups without PDA exhibited negligible temperature rises, nearly overlapping with the PBS control group. In contrast, the PDA@HAPLL group displayed significant heating, confirming that PDA served as the essential photothermal conversion agent. The Y/PDA@HAPLL group exhibited comparable photothermal conversion performance to PDA@HAPLL, reaching 50 °C within 5 min. This temperature is within the therapeutic window for effective antibacterial therapy while being below the threshold for thermal damage to mammalian cells, and the incorporation of Y-27632 did not compromise the photothermal conversion efficiency, ensuring that the composite hydrogel retained the antibacterial capability for infected wound treatment.

### 3.2. In Vitro Biocompatibility Evaluation

**Figure 2:**
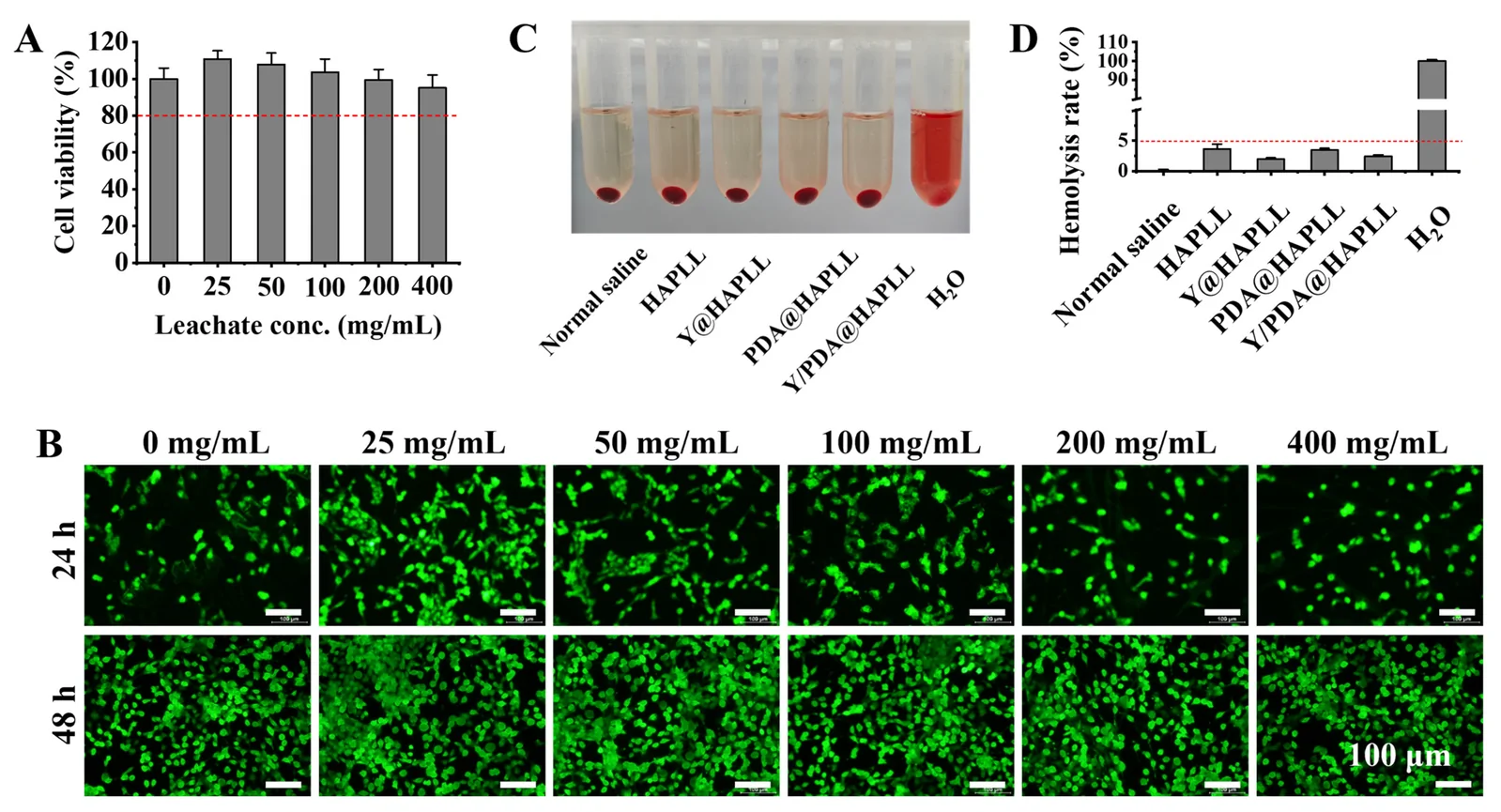
Biocompatibility evaluation of Y/PDA@HAPLL hydrogel. (**A**) MTT assay after incubation with hydrogel leachate at various concentrations (*n* = 6, $\bar{x}$ ± SD). (**B**) Live/dead cell staining fluorescence images of cells treated with different extract concentrations for 24 h and 48 h (scale bar = 100 μm). (**C**) Representative photographs of hemolysis assay for each hydrogel group; saline and water served as negative and positive controls, respectively. (**D**) Quantitative hemolysis rates of the experimental groups (*n* = 3, $\bar{x}$ ± SD).

Biocompatibility, a prerequisite for clinical translation, was evaluated according to ISO 10993-5:2009 [47] via MTT assays on NIH3T3 cells. As shown in Figure 2A, Y/PDA@HAPLL exhibited excellent cytocompatibility, with cell viability exceeding 80% at concentration up to 400 mg/mL, showing good biosafety.

Live/dead staining further visually corroborated the biocompatibility of Y/PDA@HAPLL (Figure 2B). The staining images demonstrated that viable cells (green fluorescence) were overwhelmingly predominant, while dead cells (red) were rarely observed. Furthermore, cell morphology and proliferative activity exhibited no notable differences from the blank control, indicating no significant cytotoxicity. The consistency between MTT results and live/dead observations strengthened the conclusion that Y/PDA@HAPLL does not induce appreciable cell death, which is essential for the application as a wound dressing.

According to ISO 10993-4:2017 [48], a hemolysis rate below 5% is considered acceptable for biomaterials. The in vitro hemolysis assay on Y/PDA@HAPLL (Figure 2C) showed clear supernatants similar to the saline negative control, with no obvious hemolysis. The quantitative results (Figure 2D) confirmed that all groups, including HAPLL, Y@HAPLL, PDA@HAPLL and Y/PDA@HAPLL, exhibited hemolysis rates below 5%, meeting the safety criterion of blood compatibility. Collectively, these results confirmed that the Y/PDA@HAPLL hydrogel fulfilled the basic biocompatibility and hemocompatibility requirements for the further wound healing application.

### 3.3. In Vitro Activity Evaluation of Hydrogel

#### 3.3.1. In Vitro Antibacterial Activity Assay

**Figure 3:**
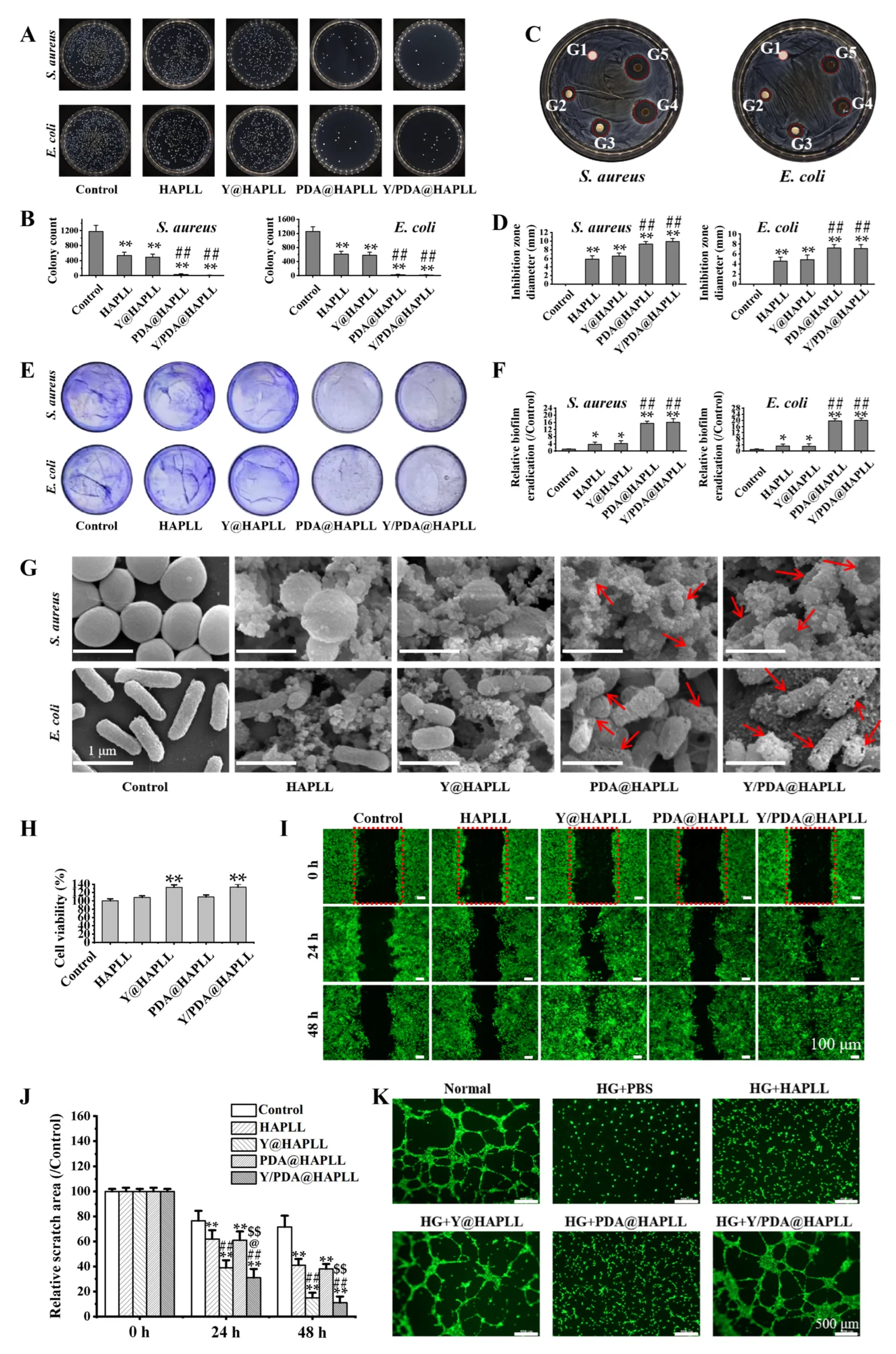
Antibacterial and wound healing performance in vitro after different treatments. (**A**) Representative colony photographs of *S. aureus* and *E. coli*. (**B**) Quantitative analysis of colony counts (*n* = 3, $\bar{x}$ ± SD), ** *p* < 0.01, compared with the control group, ^##^ *p* < 0.01, compared with the HAPLL group. (**C**) Representative images of inhibition zones against the bacterial strains, G1: Normal saline, G2: HAPLL, G3: Y@HAPLL, G4: PDA@HAPLL, G5: Y/PDA@HAPLL. (**D**) Statistical comparison of inhibition zone diameters (*n* = 3, $\bar{x}$ ± SD), ** *p* < 0.01, compared with the control group, ^##^ *p* < 0.01, compared with the HAPLL group. (**E**) Representative crystal violet staining photographs of *S. aureus* and *E. coli* biofilms. (**F**) Statistical comparison of the biofilm eradication (*n* = 3, $\bar{x}$ ± SD), * *p* < 0.05, ** *p* < 0.01, compared with the control group, ^##^ *p* < 0.01, compared with the HAPLL group. (**G**) SEM images of the bacteria, with red arrows marking the ruptured bacterial structures, scale bar = 1 μm. (**H**) Relative cell viability of HaCaT cells co-cultured with the hydrogel extracts (*n* = 6, $\bar{x}$ ± SD), ** *p* < 0.01, compared with the control group. (**I**) Fluorescence images of scratch wounds at 0, 24 and 48 h, scale bar = 100 μm. The red dashed box indicates the initial scratch area at 0 h. (**J**) Quantification of relative scratch closure area (*n* = 3, $\bar{x}$ ± SD), ** *p* < 0.01, compared with the control group, ^##^ *p* < 0.01, compared with the HAPLL group, @ *p* < 0.01, compared with the Y@HAPLL group, ^$$^ *p* < 0.01, compared with the PDA@HAPLL group. (**K**) Capillary tube formation analysis by live cell staining to assess angiogenesis, scale bar = 500 μm.

To evaluate antibacterial activity against *S. aureus* and *E. coli*, colony formation (Figure 3A,B) and zone-of-inhibition (Figure 3C,D) assays were performed. HAPLL and Y@HAPLL significantly reduced colony counts and produced clear inhibition zones versus the control, confirming the intrinsic antibacterial property of ε-PLL [49]. Under 808 nm laser irradiation, PDA-containing hydrogels (PDA@HAPLL and Y/PDA@HAPLL) exhibited markedly stronger inhibition than non-irradiated groups, owing to PDA-mediated photothermal hyperthermia that disrupts bacterial integrity. The combined action of ε-PLL and photothermal effect achieved >99% killing of both strains.

The biofilm eradication performance of the hydrogels against *S. aureus* and *E. coli* was also evaluated via a crystal violet staining assay (Figure 3E,F). HAPLL and Y@HAPLL induced only a slight reduction in biofilm biomass relative to the control, suggesting the limited intrinsic biofilm eradication activity of ε-PLL. Upon laser irradiation, PDA-containing hydrogels further augmented the capacity to eradicate the mature biofilms, as visualized by staining images and quantified by eradication efficacy calculated from absorbance values (Figure 3F). The combination of ε-PLL and the photothermal effect effectively disrupted the compact biofilm architecture. SEM images (Figure 3G) further revealed that, while controls showed intact bacterial surfaces, HAPLL and Y@HAPLL caused mild damage, whereas laser-irradiated PDA-containing groups induced severe morphological changes—*S. aureus* shrank and collapsed, and *E. coli* exhibited perforation and fragmentation—corroborating the photothermal bactericidal effect.

#### 3.3.2. In Vitro Wound Healing Assay of Hydrogels

HaCaT keratinocytes, the predominant epidermal cell type (>90%), were used to evaluate the in vitro wound healing potential of the hydrogels. The MTT results (Figure 3H) showed that Y-27632-containing groups significantly promoted keratinocyte proliferation compared with controls, indicating that Y-27632, a ROCK1 inhibitor, enhanced epidermal regeneration by modulating cytoskeletal dynamics and cell cycle progression, thereby contributing to the wound healing capacity of the hydrogel. The scratch assay (Figure 3I,J) further evaluated the wound healing effect of hydrogels on keratinocyte migration. Over time, compared with the PBS control, all hydrogel formulations significantly accelerated HaCaT cell migration. Among them, the Y/PDA@HAPLL group exhibited the fastest closure rate, with residual scratch areas at 24 h and 48 h being significantly smaller than those of the other groups. These results confirmed a combined effect between Y-27632 and PDA to effectively promote epithelial cell proliferation and migration, thereby accelerating epidermal regeneration and wound closure. The enhanced migration was likely attributable to Y-27632-mediated inactivation of ROCK1, which promoted lamellipodia formation and directional cell movement.

The tube formation assay evaluated the pro-angiogenic potential of hydrogels under high-glucose (HG) conditions (Figure 3K). The normal group exhibited abundant intact vascular networks, whereas the HG + PBS group showed severely impaired tubulogenesis, reflecting the well-established inhibitory effect of hyperglycemia on endothelial cell function. HAPLL and PDA@HAPLL slightly improved tube formation, while Y-27632-loaded groups (Y@HAPLL and Y/PDA@HAPLL) significantly increased tubular structures, with the HG + Y/PDA@HAPLL group displaying the most dense and complete network. These results confirmed that Y-27632 could effectively reverse high-glucose-induced inhibition of angiogenesis by modulating the ROCK1 pathway, which regulates endothelial cell migration and capillary-like structure formation. The promotion of angiogenesis by Y/PDA@HAPLL was expected to improve local blood supply, a critical factor for diabetic wound repair where impaired neovascularization often leads to delayed healing.

### 3.4. In Vivo Activity Evaluation of Hydrogels

#### 3.4.1. In Vivo Photothermal Conversion Performance

**Figure 4:**
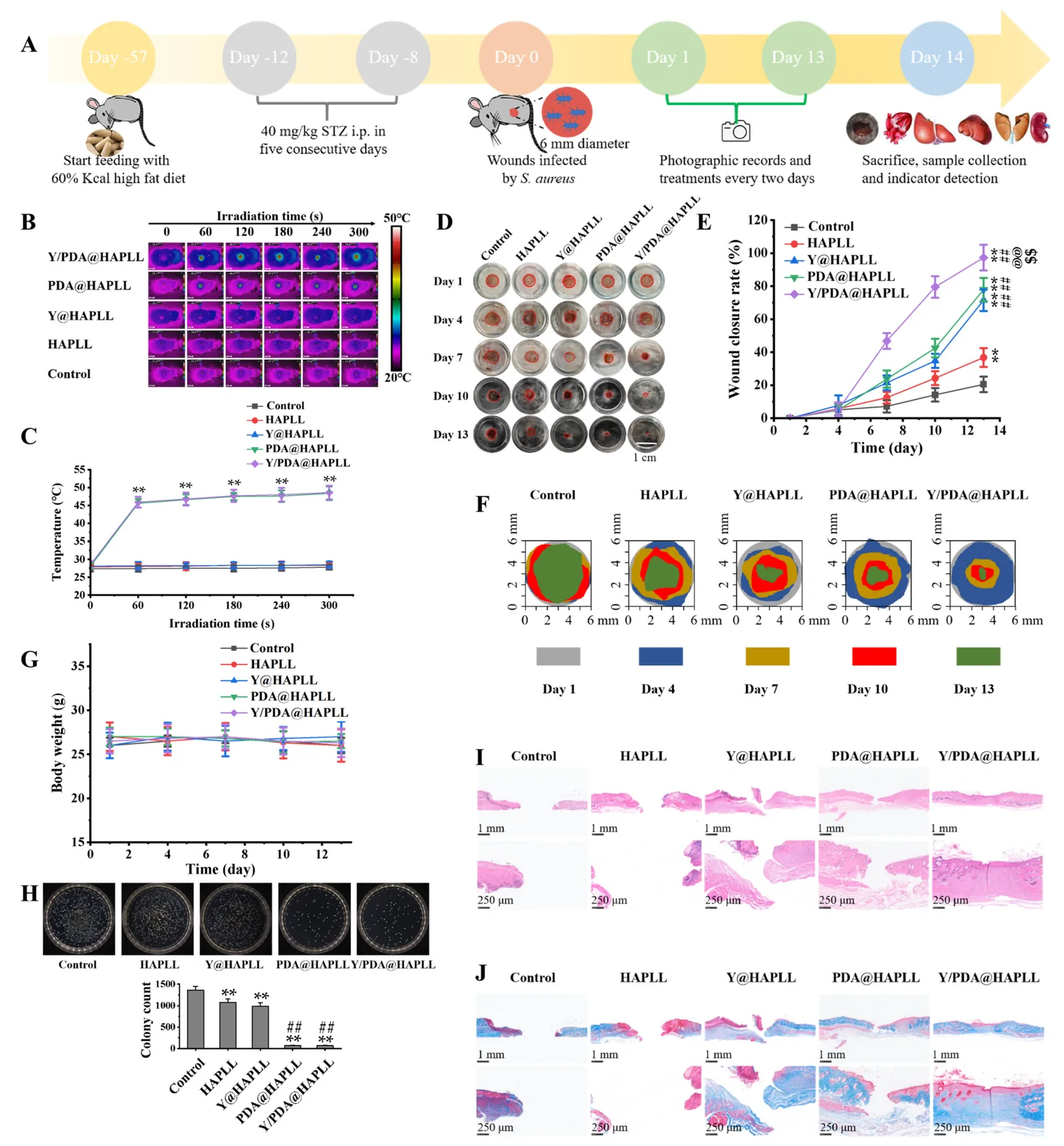
In vivo therapeutic performance of Y/PDA@HAPLL hydrogel for the diabetic wound. (**A**) Schematic establishment of the infected diabetic wound model and the treatment procedures. (**B**) Infrared thermal images of different samples under the laser irradiation. (**C**) Temperature elevation curves of each group under laser treatment (*n* = 3, $\bar{x}$ ± SD), ** *p* < 0.01, compared with the normal saline group. (**D**) Digital photographs of wound sites on day 1, 4, 7, 10 and 13, scale bar = 1 cm. (**E**) Quantitative statistics of wound closure rate within 14 days (*n* = 6, $\bar{x}$ ± SD), ** *p* < 0.01, compared with the control group, *^##^ p* < 0.01, compared with the HAPLL group, *^@@^ p* < 0.01, compared with the Y@HAPLL group, ^$$^ *p* < 0.01, compared with the PDA@HAPLL group. (**F**) Time-course images showing wound area shrinkage. The colored regions indicated the remaining wound areas, which were quantified by ImageJ software (1.51k). Scale bar = 6 mm. (**G**) Body weight changes in mice during treatments (*n* = 6, $\bar{x}$ ± SD). (**H**) Colony photos and quantitative bacterial counts of wound tissues after treatments (*n* = 3,$\bar{x}$ ± SD), ** *p* < 0.01, compared with the normal saline group, *^##^ p* < 0.01, compared with the HAPLL group. (**I**) H&E staining of wound tissues, scale bars = 1 mm (**upper**) and 250 μm (**lower**). (**J**) Masson’s trichrome staining for collagen deposition analysis in wound tissues, scale bars = 1 mm (**upper**) and 250 μm (**lower**).

The experimental timeline for diabetic wound modeling and treatment was shown in Figure 4A. To evaluate the in vivo photothermal effect, normal saline, HAPLL, Y@HAPLL, PDA@HAPLL, and Y/PDA@HAPLL were applied to dorsal wounds of mice and irradiated with the laser. As shown in Figure 4B,C, only PDA-containing hydrogels exhibited significant temperature elevation, reaching above 45 °C within 60 s and maintaining 45–50 °C over 5 min. This temperature range is highly effective for bacterial killing by inducing the thermal denaturation of membrane proteins and disruption of cell wall integrity, while causing negligible thermal damage to surrounding normal tissues due to the low thermal conductivity of skin and the rapid heat dissipation. The consistent in vivo photothermal conversion performance with in vitro results confirmed that PDA nanoparticles retained their photothermal conversion performance within the complex biological environment of wound tissue. This provided a solid foundation for in vivo antibacterial performance and ensured that the hydrogel can achieve localized hyperthermia precisely at the wound site.

#### 3.4.2. Therapeutic Effect on the Infected Diabetic Wound

The therapeutic effects are presented in Figure 4D–F. Over time, all wounds gradually contracted, but the healing effect varied significantly. The control group showed the slowest closure rate. HAPLL alone slightly accelerated wound reduction, with limited overall improvement. The Y@HAPLL and PDA@HAPLL groups exhibited markedly faster shrinkage and higher closure rates than the first two groups, indicating that Y-27632 promoted healing by modulating the ROCK1 pathway to enhance epithelial cell migration and proliferation, while PDA-mediated photothermal sterilization contributes by reducing bacterial burden that would otherwise impede wound repair. Notably, Y/PDA@HAPLL achieved the best repair outcome, with the largest wound reduction throughout and the smallest residual area on day 13, showing statistically significant differences versus all other groups. Time-course images further confirmed the combined effect between Y-27632 and PDA, where the former drove cellular regeneration and the latter created a sterile environment conducive to healing. Additionally, body weight remained largely unaffected in all hydrogel-treated groups (Figure 4G). In summary, Y/PDA@HAPLL hydrogel significantly accelerated the wound healing, demonstrated remarkable potential for treating the infected diabetic wound.

The wound bacterial colonies were counted (Figure 4H), and all the hydrogel groups showed significantly fewer colonies than the control due to the ε-PLL’s intrinsic antibacterial activity. PDA@HAPLL and Y/PDA@HAPLL had the lowest counts, confirmed that the PDA-mediated photothermal effect eliminated wound bacteria. H&E staining of wound tissues (Figure 4I) revealed that the saline group had wide gaps, severe inflammation, and minimal new tissue, reflecting the impaired healing cascade in diabetic conditions. HAPLL slightly improved repair by providing a moist wound environment and mild antimicrobial action. Y@HAPLL and PDA@HAPLL showed better closure but incomplete epithelium, indicating that either the ROCK1 inhibitor or photothermal sterilization alone partially addressed the complex pathology of diabetic wound. Y/PDA@HAPLL achieved the best outcome: nearly complete bridging, continuous epidermis, reduced inflammatory cells, abundant collagen and neovessels, and mature granulation tissue, confirming that Y-27632 and PDA jointly enhanced anti-inflammation, epithelialization, and tissue remodeling.

Masson staining (Figure 4J) showed the saline group had large wound gaps with sparse blue collagen, indicating poor synthesis and disorganized deposition. HAPLL slightly increased collagen but with obvious defects reflecting insufficient structural support. Y@HAPLL and PDA@HAPLL exhibited greater deposition but disorganized arrangement, suggesting that while regeneration was stimulated, the quality of repair tissue remained suboptimal. Y/PDA@HAPLL achieved nearly closed wounds with uniformly stained, well-aligned collagen fibers in the dermis, demonstrating superior remodeling that closely resembles normal skin architecture. These results confirmed that Y-27632 and PDA co-modified Y/PDA@HAPLL could significantly promote collagen synthesis and accelerate skin tissue remodeling, likely through a combination of enhanced fibroblast activity, reduced inflammation, and improved angiogenesis. The well-aligned collagen fibers in the Y/PDA@HAPLL group indicated that the Y/PDA@HAPLL hydrogel not only accelerated healing but also improved the quality of the regenerated tissue, which is essential for functional recovery of the diabetic wound.

**Figure 5:**
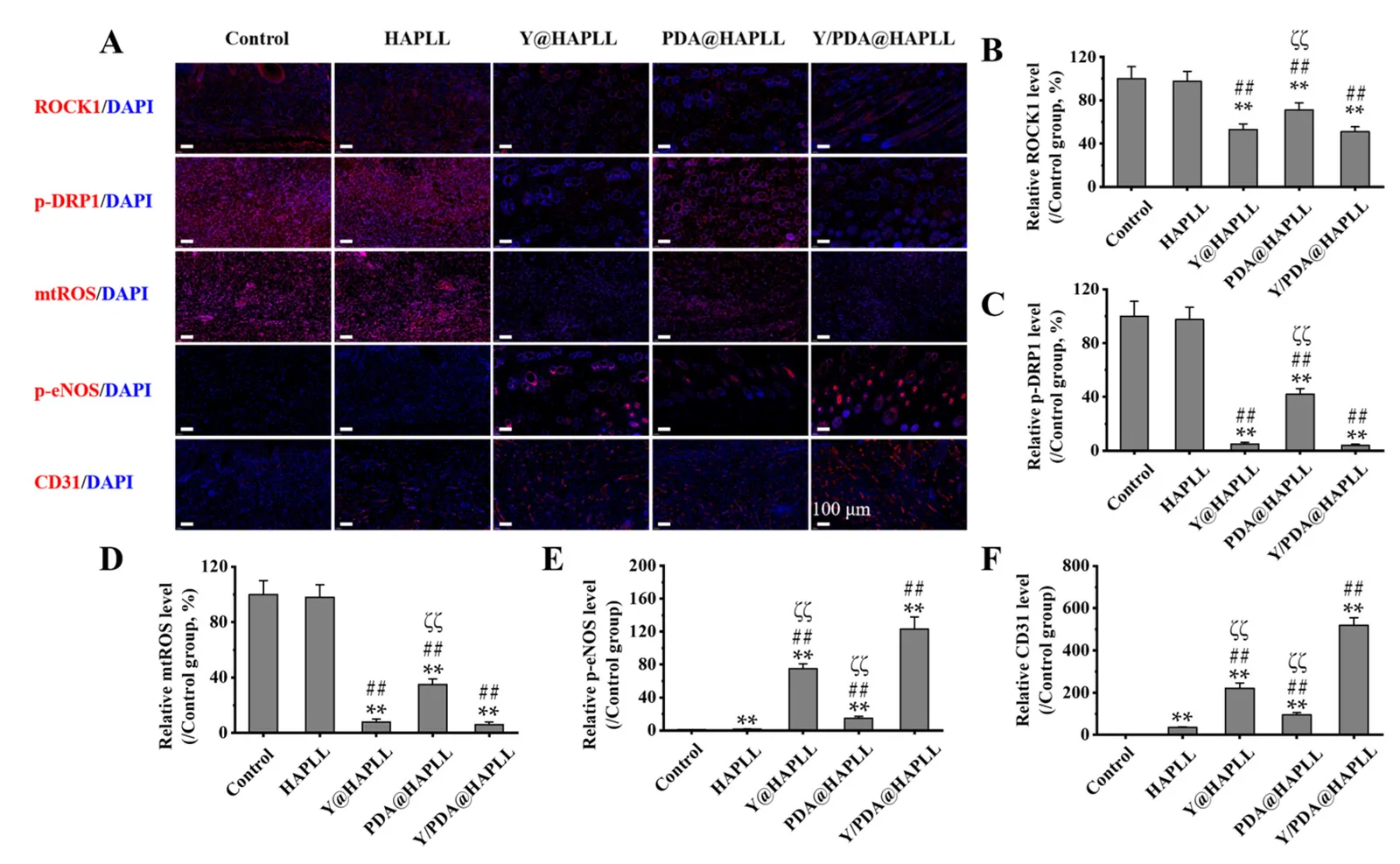
The therapeutic effect of Y/PDA@HAPLL hydrogel on regulating ROCK1/p-DRP1 pathway and angiogenesis in wound. (**A**) Immunofluorescence staining images of ROCK1, p-DRP1, mtROS, p-eNOS and CD31 in wound sections, scale bar = 100 μm. (**B**) Quantitative analysis of relative ROCK1 fluorescence intensity (*n* = 3, $\bar{x}$ ± SD), ** *p* < 0.01, compared with the control group, ^##^ *p* < 0.01, compared with the HAPLL group, ^ζζ^ *p* < 0.01, compared with the Y/PDA@HAPLL group. (**C**) Statistical quantification of relative p-DRP1 fluorescence level (*n* = 3, $\bar{x}$ ± SD), ** *p* < 0.01, compared with the control group, ^##^ *p* < 0.01, compared with the HAPLL group, ^ζζ^ *p* < 0.01, compared with the Y/PDA@HAPLL group. (**D**) Relative mtROS fluorescence intensity statistics (*n* = 3, $\bar{x}$ ± SD), ** *p* < 0.01, compared with the control group, ^##^ *p* < 0.01, compared with the HAPLL group, ^ζζ^ *p* < 0.01, compared with the Y/PDA@HAPLL group. (**E**) Quantitative results of relative p-eNOS expression level (*n* = 3, $\bar{x}$ ± SD), ** *p* < 0.01, compared with the control group, ^##^ *p* < 0.01, compared with the HAPLL group, ^ζζ^ *p* < 0.01, compared with the Y/PDA@HAPLL group. (**F**) Relative CD31 fluorescence intensity (*n* = 3, $\bar{x}$ ± SD), ** *p* < 0.01, compared with the control group, ^##^ *p* < 0.01, compared with the HAPLL group, ^ζζ^ *p* < 0.01, compared with the Y/PDA@HAPLL group.

The therapeutic effect was also elucidated by immunofluorescence staining of wound tissues (Figure 5A). Compared with the saline and HAPLL groups, the Y@HAPLL, PDA@HAPLL, and Y/PDA@HAPLL groups significantly downregulated ROCK1 expression (Figure 5B), with the Y/PDA@HAPLL group showing the strongest inhibition, thereby blocking downstream damage pathways and stabilizing cellular states. This ROCK1 downregulation is attributed to the release of Y-27632, a selective ROCK1 inhibitor, which disrupted the RhoA/ROCK1 signaling cascade that was often hyperactivated in diabetic wound and contributed to impaired cell migration and excessive inflammation. Similarly, p-DRP1 expression (Figure 5C), a marker of mitochondrial fission, was markedly reduced in these groups, indicating the alleviation of excessive mitochondrial fragmentation and improved cellular homeostasis. The reduction in p-DRP1 suggested that ROCK1 inhibition attenuated Drp1 phosphorylation, thereby limiting mitochondrial fission and preserving mitochondrial network integrity, which was crucial for maintaining energy metabolism and preventing apoptosis in wound-edge cells. Moreover, mtROS levels (Figure 5D) were substantially decreased, confirming that the co-modification effectively scavenged mitochondrial reactive oxygen species and attenuates oxidative stress. The decreased mtROS production likely resulted from both the reduced mitochondrial fission and the direct antioxidant properties of PDA nanoparticles, which can scavenge the free radicals, thereby breaking the vicious cycle of oxidative damage and mitochondrial dysfunction that perpetuated delayed healing in diabetic wounds. Concurrently, p-eNOS expression (Figure 5E) was significantly upregulated in Y-27632-containing groups, promoting nitric oxide production to enhance angiogenesis and microcirculation. This upregulation reflected the well-established role of ROCK1 inhibition in restoring eNOS phosphorylation and activity, which was often suppressed under hyperglycemic conditions, leading to improved endothelial function and vasodilation. CD31 staining (Figure 5F) further demonstrated that Y/PDA@HAPLL induced the highest neovascularization among all groups, confirming that enhanced eNOS activity translated into functional angiogenesis. Collectively, these combined effects—suppression of pro-inflammatory and pro-fission signals, reduction in oxidative damage, and promotion of endothelial function—optimized the wound microenvironment, accelerated epithelialization, collagen remodeling, and granulation maturation, thereby significantly enhancing diabetic wound repair. The comprehensive modulation of these interrelated pathways by the Y/PDA@HAPLL hydrogel highlighted its unique advantage as a multifunctional platform that addressed the complex pathophysiology of diabetic wounds, rather than targeting a single pathogenic mechanism.

**Figure 6:**
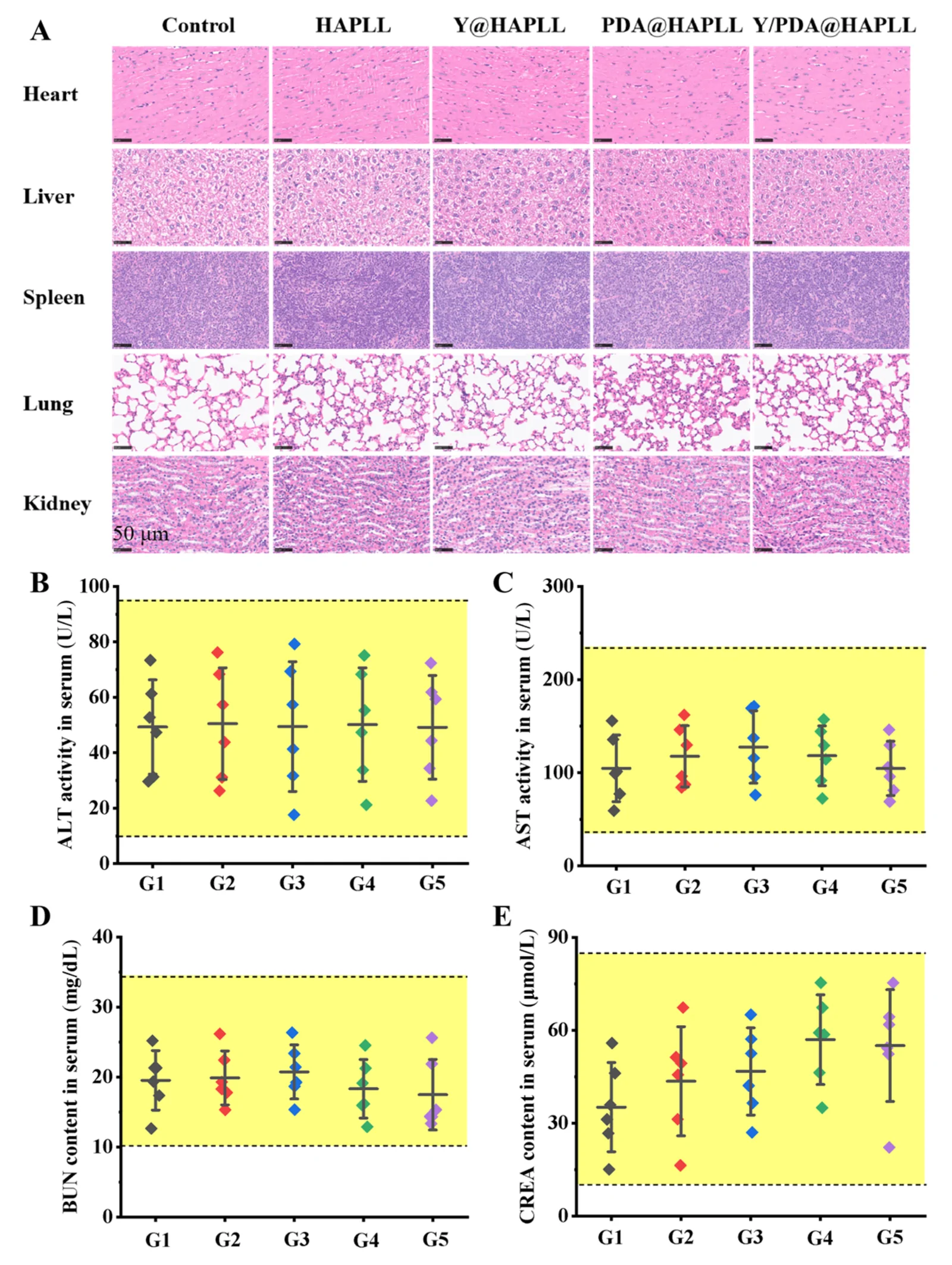
The systematic effect on major organs after different treatments. (**A**) H&E staining images of the major organs, scale bar = 50 μm. (**B**–**E**) Serum biochemical indicators including ALT, AST, BUN and CREA of mice (*n* = 6, $\bar{x}$ ± SD), light-yellow area: the normal physiological range. G1: Control, G2: HAPLL, G3: Y@HAPLL, G4: PDA@HAPLL, G5: Y/PDA@HAPLL.

H&E staining of the major organs (Figure 6A) showed no pathological abnormalities in heart, liver, spleen, lung, and kidney tissues after different treatments. The major organs exhibited normal histological features: myocardial fibers were regularly aligned, hepatic lobules remained intact, splenic cells were evenly distributed, alveolar architecture was normal, and renal tubules showed no edema, necrosis, or inflammatory infiltration. Serum ALT, AST, BUN, and CREA were also measured to assess the hepatic and renal function (Figure 6B–E). All values for Control, HAPLL, Y@HAPLL, PDA@HAPLL, and Y/PDA@HAPLL groups were within normal reference ranges. The treatments did not cause significant systematic toxicity in vivo.

## 4. Conclusions

In summary, a Schiff-base crosslinked stretchable multifunctional Y/PDA@HAPLL hydrogel was successfully engineered for infected diabetic wound management. The formulation was rationally optimized via response surface methodology, achieving rapid in situ gelation (3.8 s), robust tissue adhesion (10 kPa). The hydrogel exhibited a uniform interconnected porous network, excellent stretchability, pH-responsive drug release and stable photothermal conversion performance under an 808 nm laser, enabling a combined antibacterial and antibiofilm efficacy via ε-PLL’s intrinsic activity and the PDA-mediated photothermal effect, which achieved >99% killing of *S. aureus* and *E. coli*, as confirmed by zone of inhibition and colony assays. In vitro studies revealed outstanding cytocompatibility, hemocompatibility, and wound healing functions, including enhanced skin re-epithelialization (keratinocyte proliferation and migration) and angiogenesis under high glucose via Y-27632. In vivo, the hydrogel effectively cleared wound bacteria and accelerated diabetic wound closure, facilitated tissue remodeling, downregulated the expression of ROCK1 and p-DRP1, reduced mitochondrial ROS (mtROS) levels, and upregulated p-eNOS and CD31 expression. Importantly, no significant hepatorenal toxicity or organ damages were detected, confirming excellent systematic biosafety. Collectively, the Y/PDA@HAPLL hydrogel represented a promising multifunctional platform for the infected wound repair, combining rapid gelation, photothermal antibacterial activity, and wound healing capacity, with the promising potential for clinical translation in diabetic wound therapy.

## Data Availability

The data that support the findings of this study are available from the corresponding authors upon reasonable request.

## Supplementary Materials

The following supporting information can be downloaded at: https://www.mdpi.com/article/10.3390/jfb17100493/s1, Figure S1: ^1^H NMR spectra and chemical structure. (A) HA. (B) OHA; Figure S2: Preparation of polydopamine (PDA) nanoparticles; Figure S3: Particle size distribution of PDA nanoparticles; Figure S4: Microscopic morphologies of PDA nanoparticles. (A) TEM image. (B) SEM image. Scale bar = 100 nm; Figure S5: Temperature rise curves under 808 nm laser with diverse power densities (n = 3, $\bar{x}$ ± SD); Table S1: Factors and corresponding levels used in the experimental design; Table S2: The experiments and responses for Y/PDA@HAPLL hydrogel; Table S3: Analysis of variance (ANOVA) of regression model with gelation time as the response value; Table S4: Analysis of variance (ANOVA) of regression model with adhesion strength as the response value; Table S5: Comparison between predicted and experimental values under optimal concentrations (19.35% OHA and 22.29% ε-PLL).

## Abbreviations

The following abbreviations are used in this manuscript:

OHA	Oxidized hyaluronic acid
AMPKα	Adenosine 5′-monophosphate (AMP)-activated protein kinase
ε-PLL	ε-poly-L-lysine
Y	Y-27632
PDA	Polydopamine
ROS	Reactive oxygen species
p-eNOS	Phosphorylated endothelial-type nitric oxide synthase
ROCK1	Rho-associated coiled-coil containing protein kinase 1
p-DRP1	Phosphorylated dynamin-related protein 1
NIR	Near-infrared
mtROS	Mitochondrial reactive oxygen species
HA	Hyaluronic acid
RT	Room temperature
PTT	Photothermal therapy
TEM	Transmission electron microscopy
SEM	Scanning electron microscopy
DLS	Dynamic light scattering
DSP	Disodium hydrogen phosphate
RSM	Response surface methodology
CCD	Central composite design
FESEM	Field emission scanning electron microscopy
PBS	Phosphate-buffered saline
HPLC	High performance liquid chromatography
TFA	Trifluoroacetic acid
FBS	Fetal bovine serum
P/S	Penicillin/streptomycin
MTT	3-(4,5-dimethylthiazol-2-yl)-2,5-diphenyltetrazolium bromide
DMSO	Dimethyl sulfoxide
LB	Luria–Bertani
STZ	Streptozotocin
FBG	Fasting blood glucose
H&E	Hematoxylin-eosin
RBCs	Rat red blood cells
