# Supplementary material for: Schiff-Base Crosslinked Stretchable Multifunctional Hydrogel Enables Combined Therapy in Diabetic Wound Healing
Source: J Funct Biomater. 2026 Sep 30;17(10):493. doi: 10.3390/jfb17100493 (PMC13705685; doi:10.3390/jfb17100493)
Supplement: Supplementary file 1 [file jfb-17-00493-s001.zip › jfb-4536291-supplementary.pdf]

Article

Schiff-Base Crosslinked Stretchable Multifunctional Hydrogel Enables Combined Therapy in Diabetic Wound Healing

Ze-Dong Cai [1,†], Yao-Wen Zhang [1,†], Xin-Ru Zou [1], Jia Ju [2,*], Chao Fang [3], Ming-Kai Li [3] and Bang-Le Zhang [1,*]

[1] Department of Pharmaceutics, School of Pharmacy, and Key Laboratory of Pharmacology of the State
Administration of Traditional Chinese Medicine, Fourth Military Medical University, Xi'an 710032, China; pharmczd@163.com (Z.-D.C.); z17860554048@163.com (Y.-W.Z.); zxr01092024@163.com (X.-R.Z.)

[2] Department of Pharmacy, School of Stomatology, Fourth Military Medical University, Xi'an 710032, China

[3] Department of Pharmacology, School of Pharmacy, Fourth Military Medical University, Xi'an 710032, China; fang1989@fmmu.edu.cn (C.F.); mingkai@fmmu.edu.cn (M.-K.L.)

* Correspondence: jujia143@163.com (J.J.); blezhang@fmmu.edu.cn (B.-L.Z.)

† These authors contributed equally to this work.

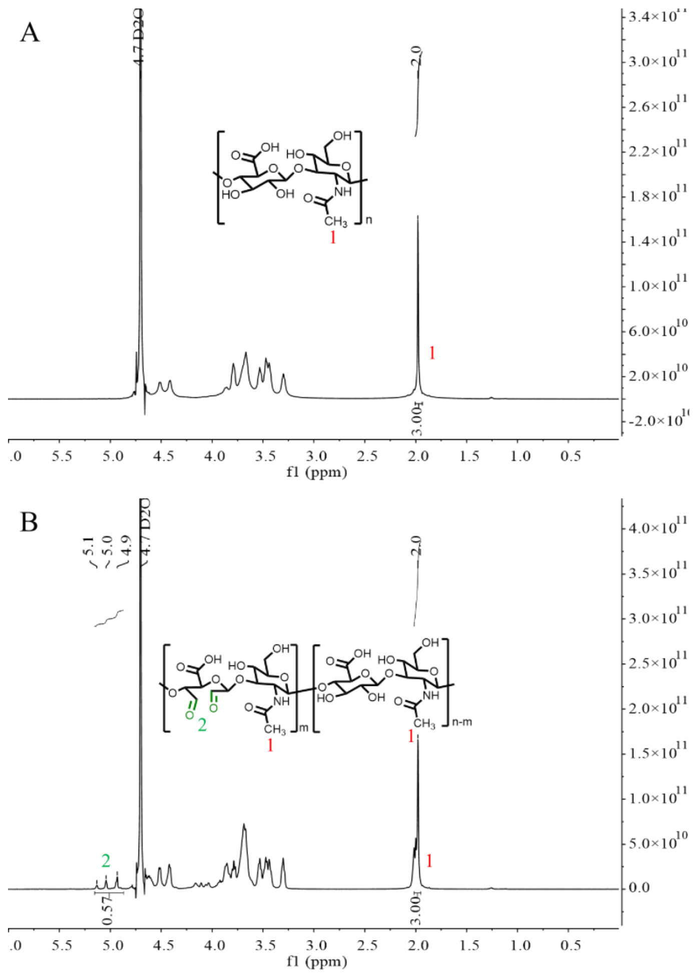

Figure S1. ^{1}H NMR spectra and chemical structure. (A) HA. (B) OHA

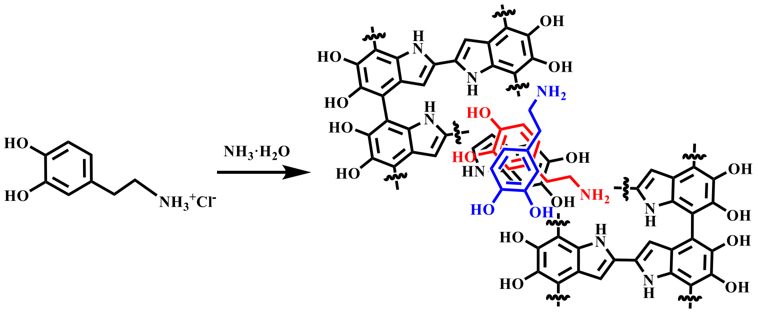

Figure S2. Preparation of polydopamine (PDA) nanoparticles

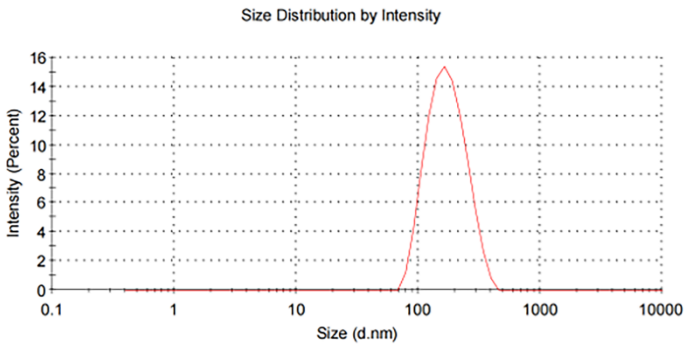

Figure S3. Particle size distribution of PDA nanoparticles

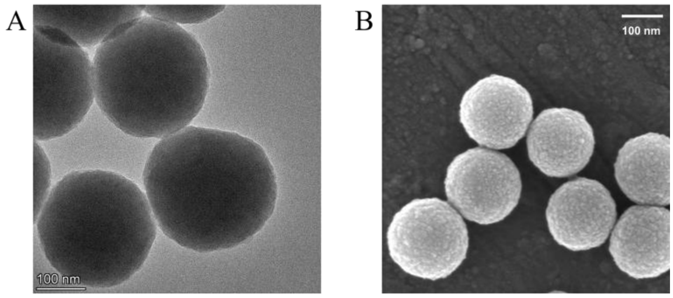

Figure S4. Microscopic morphologies of PDA nanoparticles. (A) TEM image. (B) SEM image. Scale bar = 100 nm

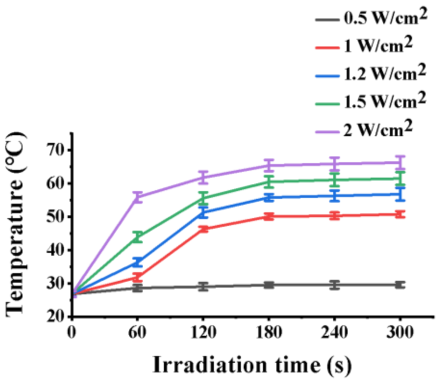

Figure S5. Temperature rise curves under 808 nm laser with diverse power densities (n = 3, $\bar{x}$ ± SD)

Table S1. Factors and corresponding levels used in the experimental design

Factor	Code	The Level of Code		
		−1	0	1
OHA (w/v, %)	A	10	15	20
PLL (w/v, %)	B	10	25	40

Footnote: The concentrations of OHA and PLL were the initial concentrations before mixing.

Table S2. The experiments and responses for Y/PDA@HAPLL hydrogel

Std	Run	Factor 1	Factor 2	Response 1	Response 2
		A: OHA (w/v, %)	B: PLL (w/v, %)	Gelation time (s)	Adhesion strength (kPa)
13	1	15	25	7	9.75
6	2	20	25	4	11.25
5	3	10	25	29	4.25
3	4	10	40	48	1.5
10	5	15	25	12	8.75
8	6	15	40	25	6.75
1	7	10	10	32	2.75
2	8	20	10	11	9.25
11	9	15	25	10	9.5
4	10	20	40	19	7.5
9	11	15	25	9	10
7	12	15	10	15	8
12	13	15	25	11	9

Table S3. Analysis of variance (ANOVA) of regression model with gelation time as the response value

Source	Sum of Squares	df	Mean Square	F-value	p-value
Model	1855.12	5	371.02	156.69	< 0.0001 significant
A - OHA	937.5	1	937.5	395.93	< 0.0001
B - PLL	192.67	1	192.67	81.37	< 0.0001
AB	16	1	16	6.76	0.0355
A^2	138.69	1	138.69	58.57	0.0001

B^2	309.52	1	309.52	130.72	< 0.0001	
Residual	16.57	7	2.37			
Lack of Fit	1.77	3	0.5916	0.1599	0.918	not significant
Pure Error	14.8	4	3.7			
Cor Total	1871.69	12				
	Std. Dev. = 1.54; R^2 = 0.9911					
	Mean = 17.85; Adjusted R^2 = 0.9848					
	C.V. % = 8.62; Predicted R^2 = 0.9796					
	Adeq Precision = 41.3749					

Table S4. Analysis of variance (ANOVA) of regression model with adhesion strength as the response value

**Source**	**Sum of Squares**	**df**	**Mean Square**	***F*-value**	**p-value**	
Model	104.71	5	20.94	101.87	< 0.0001	significant
A - OHA	63.37	1	63.37	308.3	< 0.0001	
B - PLL	3.01	1	3.01	14.64	0.0065	
AB	0.0625	1	0.0625	0.304	0.5985	
A^2	9.75	1	9.75	47.45	0.0002	
B^2	14.04	1	14.04	68.28	< 0.0001	
Residual	1.44	7	0.2056			
Lack of Fit	0.3639	3	0.1213	0.4514	0.7303	not significant
Pure Error	1.07	4	0.2687			
Cor Total	106.14	12				
	Std. Dev. = 0.4534; R^2 = 0.9864					
	Mean = 7.56; Adjusted R^2 = 0.9768					
	C.V. % = 6.00; Predicted R^2 = 0.9552					
	Adeq Precision = 30.3153					

Table S5. Comparison between predicted and experimental values under optimal concentrations (19.35% OHA and 22.29% ε-PLL)

**Response**	**Predicted**	**Experimental value**	***p*-value**	
Gelation time (s)	3.81	3.77 ± 0.47	$p > 0.05$	not significant
Adhesion strength (kPa)	10.95	10.08±2.02	$p > 0.05$	not significant
